# The Role of Digital Maturity in Achieving the Quality of E-Services: An Analytical Study at Al-Rafidain Bank – Fallujah Branch

**DOI:** 10.12688/f1000research.174203.2

**Published:** 2026-07-27

**Authors:** Younis Akram Turki, Zaid Khawam Mahmood

**Affiliations:** 1 Public Administration, University of Fallujah, Al-Fallujah, Al Anbar Governorate, 31000, Iraq

**Keywords:** Keywords: Digital Maturity, E Service Quality, Al-Rafidain Bank.

## Abstract

**Objective:**

The study aims to explore how the features and components of digital maturity influence the enhancement of e-service quality at Al-Rafidain Bank – Fallujah Branch.

**Design/Methodology:**

The study employed a descriptive-analytical methodology, utilizing a questionnaire as the primary tool to measure the relationships between variables.

**Theoretical/Framework:**

This study is underpinned by the Extended Unified Theory of Acceptance and Use of Technology (UTAUT2) as a theoretical framework to explain the relationship between digital maturity and e-service quality. This study investigates the impact of digital maturity on e-service quality (reliability, trust, security, responsiveness, ease of use, data confidentiality, etc.) to find out if Al-Rafidain Bank will be able to serve its customers more efficiently and effectively in the coming years.

**Findings:**

The findings showed a strong correlation and a significant impact of digital maturity on the quality of e-services offered. It was also established that digital maturity explained between 53% and 56% of the service quality variance. The strongest drivers in this regard included technological culture, organizational structures, and visions and perceptions. These findings suggest that to achieve superior quality in e-services, having technology available is a prerequisite. However, having technology is not enough; it should be combined with organizational elements, staff empowerment, and a predominant digital vision. Thus, digital maturity is an essential determinant for performance improvement and institutional digital transformation.

**Conclusions:**

The results showed that Al-Rafidain Bank – Fallujah Branch, Al-Rafidain Bank in the Fallujah branch, maintains a high level of digital maturity, which is a result of advanced technology coupled with quality e-services. The technological culture organization, and digital vision framework uniquely enhance service quality, demonstrating the need to provide all the digital components in order to attain a tangible transformation that is viable and efficient.

## Introduction

The interest of scholars regarding the use of information technology (IT) for management purposes began in the 1950s, following the development of the ENIAC computer in 1945 and subsequently, the first computer in 1945 ( Gao & Yanjun, 2022: 45). The revolutionary shift in the Internet during the 1990s resulted in a dramatic change in the storage of documents, moving from paper to digital formatting and practicing the process of digitizing information ( Alhamamsheh, 2018: 5). Organizations tend to gradually improve digital transformation by focusing on foundational transformation, which is termed digital maturity. The digital maturity model introduced for the first time in 2011 has attracted a great deal of interest, although it is still subject to debate regarding its various models ( Tonder et al., 2024: 111). The current measure of earned value for a project in an organization complex structures serves as a strategy for optimum resource utilization ( Rodriguez et al., 2024: 3). To overcome the numerous challenges posed by rapid technological change, digital maturity is critical ( Al Qudah, 2024: 3). Practically, all services, banking services, have been digitized due to the evolution of Industry 4.0.

Digital banks have emerged in response to rapid customer demand for enhanced access and seamless service options ( Utama & Trisnawati, 2024: 174). Through Haryanti’s model, a guide for digital transformation in institutions is articulated in terms of developmental stages ( Haryanti, 2023: 4). Steps toward success include both hydraulic and management systems ( Cosa & Torelli, 2024: 454). With advances in digital technology and the increase in opportunities created by technological innovation, the banking industry is one of the many industries in the world that is changing and improving in technology, which helps improve customer satisfaction and overall service quality ( Lumbantobing et al., 2025: 1935). With the increase in online technology, customers have more unrealistic expectations and become less forgiving about bad services ( Kamkar et al., 2023: 2). With technological advancements, the processes and marketing of services by banks are simplified, reducing costs and time ( Pourmohammad et al., 2015: 522). Hence, the primary strategy to accumulate more knowledge of digital technology is constructive in the development of modern banking services ( Elkhaldi & Abdullah, 2022: 2).

This study adopts the (UTAUT2) model as a widely used framework in technology acceptance research, due to its ability to explain user behavior toward adopting modern technologies ( Schett & Hampl, 2025: 383). The (UTAUT2) model incorporates additional variables such as hedonic motivation, perceived price value, and habit, thereby providing a broader explanation of technology acceptance. This becomes particularly important when technologies function as interactive and engaging tools that contribute to identity formation, rather than merely serving as functional systems ( Aras, 2026: 3). The field of technology acceptance has undergone rapid developments since the emergence of its first theoretical framework, namely the Diffusion of Innovations theory in 1962. These developments are reflected in the diversification of technology acceptance indicators that have been incorporated and refined within multiple models over the years ( Kułak et al., 2019: 102). The Unified Theory of Acceptance and Use of Technology (UTAUT2) is an extended version introduced by Viswanath Venkatesh and colleagues (2012), based on the original (UTAUT) model, with a particular focus on explaining consumer behavior in technology acceptance ( Wu & Zhang, 2026: 2). This model expands the original framework by adding three key variables: perceived value (PV), hedonic motivation (HM), and habit (H), while retaining the core variables of performance expectancy (PE), effort expectancy (EE), and social influence (SI) ( Or, 2026: 54). Although the original UTAUT model effectively explains organizational technology adoption, Viswanath Venkatesh and colleagues (2012) extended it into UTAUT2 to better account for consumer technology acceptance, incorporating external variables such as hedonic motivation, habit, and perceived price value ( Acikgul & Sad, 2026: 3).

According to ( Kułak et al., 2019: 102), the Unified Theory of Acceptance and Use of Technology has evolved over the years as follows:

1. Diffusion of Innovations Theory (IDT) – 1962
2. Theory of Reasoned Action (TRA) – 1967
3. Model of PC Utilization (MPCU) – 1979
4. Theory of Planned Behavior (TPB) – 1985
5. Social Cognitive Theory (SCT) – 1986
6. Technology Acceptance Model (TAM) – 1986
7. Motivational Model (MM) – 1992
8. Combined Model (C-TAM-TPB) – 1995
9. Unified Theory of Acceptance and Use of Technology (UTAUT) – 2003
10. Unified Theory of Acceptance and Use of Technology 2 (UTAUT2) – 2012

Finally, the Unified Theory of Acceptance and Use of Technology (UTAUT2) is widely employed to explain both consumer and organizational adoption of digital technologies ( Gamit & Jhala, 2026: 27).

## Section one: General framework and methodology of the study

### 1. Research problem

As a result of the changes taking place in the digital ecosystem and the increasing adoption of technology in service delivery, banks face a pressing problem: the development of digital systems with the components of quality and efficiency.

One modern concept, echoing digital transformation within a system, is “Digital Maturity.” This indicates the effectiveness of a system in optimizing its resources, particularly in terms of the quality of e-services. Despite technical progress, there is a digital service gap in several Iraqi banks. This narrows down to the level of the e-services rendered. This raises pertinent issues regarding the digital service gap and its service components, such as service speed, service accuracy, service security, and customer satisfaction, This study addresses critical operational challenges at Al-Rafidain Bank – Fallujah Branch, where technology adoption lacks organizational readiness and efficiency. Specifically, this gap manifests in frequent system downtime (undermining reliability and security), slow digital response times for customer complaints, and low customer adoption of mobile applications due to perceived complexity. Consequently, the core research problem lies in investigating how “Digital Maturity” (Technological Culture, Technological Tools, Organizational Structures, Visions and Perceptions) can act as a strategic driver to bridge these gaps and enhance e-service quality dimensions—such as reliability, trust, security, responsiveness, ease of use, data confidentiality—transforming platforms into secure, customer-centric solutions.

Thus, the research issue arises from the primary question:

“What is the impact of digital maturity on the quality of e-services at Al-Rafidain Bank – Fallujah Branch?”

This primary question splits into several sub-questions, such as:

(a) “Which level of digital maturity is exhibited at Al-Rafidain Bank – Fallujah Branch?”
(b) “How would you assess the quality of e-services offered by the branch?”
(c) “What is the impact of digital maturity on the quality of those services?”
(d) “What barriers does the bank encounter in advancing its digital maturity?”

### 2. Research significance

**Figure 1.**
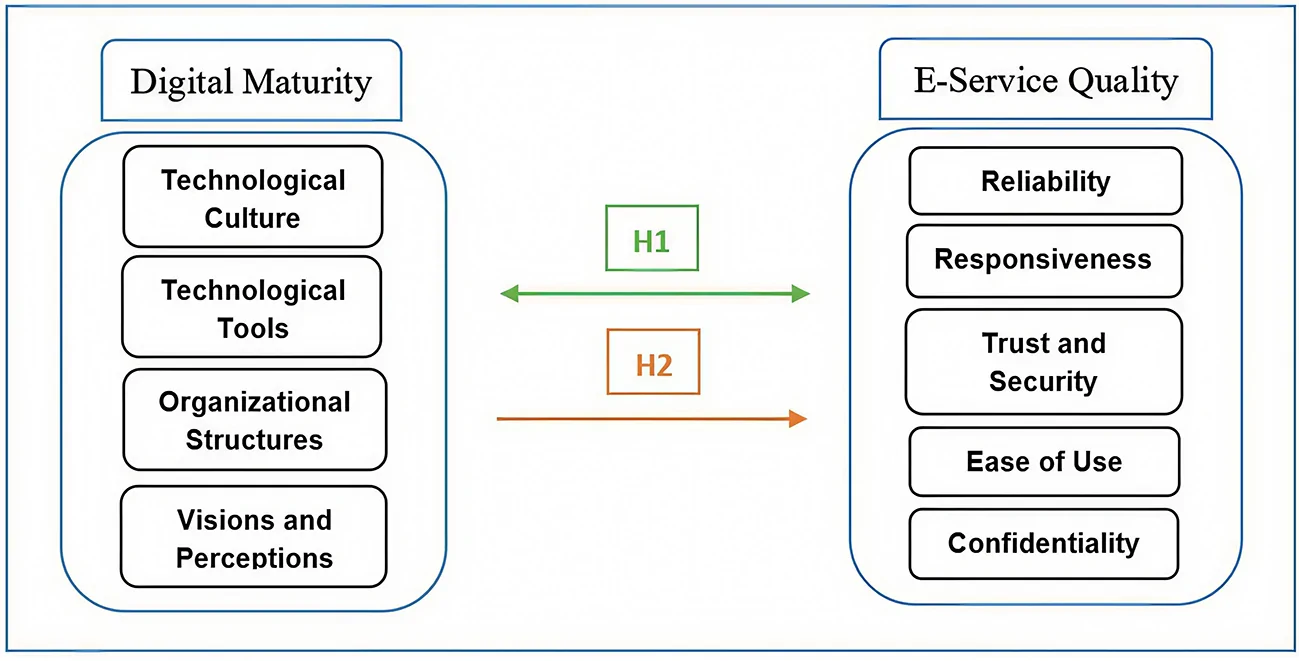
The hypothetical research framework.

The relevance of this research is reflected in two dimensions:

1. **Academic Significance:**
  (a) By applying these concepts to the Iraqi banking sector, this study increases the body of work on the intersection of digital maturity and e-service quality.
  (b) Treating digital maturity as an important variable sets the stage for further research on the maturity level of financial services.
  (c) This provides an analytical framework that can be utilized in other environments through subsequent research.
2. **Practical Significance:**
  (a) This research helps Al-Rafidain Bank managers assess the branch’s level of digital maturity and interpret the available data and analysis to solve problems.
  (b) This analysis provides a comparative assessment of electronic e-services, which contributes to the enhancement of customer experience and customer satisfaction.
  (c) Other banks in Iraq can use this study to understand digital transformation strategies and how to implement them.
3. **Research Objectives:**
  This study intends to achieve the following objectives:
  (a) Assess the level of digital maturity of Al-Rafidain Bank – Fallujah Branch.
  (b) Assess the e-service quality offered by the branch using the service quality dimensions of reliability, responsiveness, trust and security, ease of service use, and confidentiality.
  (c) The correlation between digital maturity and e-service quality as well as the degree to which the former improves the latter.
  (d) Rationalize necessary practical suggestions for the bank to electronically develop its services to improve the service quality offered to its customers.
4. **Restating the hypotheses and underlying the frameworks:**
  Through an extensive review of the literature on digital maturity along with E-Service Quality and the elements that underpin the two, the research framework shown in Figure 1 has been hypothesized. This piece of structure has been developed with regard to the research problem along with its objectives. The primary research outcomes were as follows:
1. **Main Hypothesis One (H1)**
  “There is a correlation that is statistically significant, and that relates to the dimensions of digital maturity, as well as the e-service’s overall quality.”
  This has spawned four sub-hypotheses, which are:
  (a) There exists a statistically significant level of correlation between the dimension of culture and the quality of e-services.
  (b) There exists a statistically significant level of correlation between the dimensions of technological tools and the quality of e-services.
  (c) There is a level of correlation that is statistically significant between the dimension of organizational structures and the quality of e-services.
  (d) There exists a level of correlation that is statistically significant between the dimensions of visions and perceptions and the quality of e-services.
2. **Main Hypothesis Two (H2)**
  “There exists a level of correlation that is statistically significant, and that relates to digital maturity, as well as the overall quality of electronic services.”
  This has also spawned four sub-hypotheses, which are:
  (a) The technological culture dimension had a statistically significant impact on the quality of e-services.
  (b) The use of technological tools has been found to significantly impact the quality of e–services.
  (c) The use of organizational structures has been found to significantly impact the quality of e–services.
  (d) The use of vision and perception have been noted to significantly impact the quality of e–services.

## Research Instrument: Design/Methodology and Validation

The primary data collection tool is a structured self-administered questionnaire. The instrument was developed based on established scales in the literature, specifically aligned with the UTAUT2 framework and ISO standards for e-service quality. To ensure face validity, the initial draft was reviewed by a panel of academic experts (arbitrators) specializing in Management Information Systems (MIS) and Public Administration. Their feedback regarding clarity, relevance, and technical phrasing was incorporated before final distribution. Furthermore, a pilot study was conducted to confirm the instrument's reliability through Cronbach’s Alpha and Split-Half coefficients. justified by its efficacy in examining causal relationships within structured banking environments. Data were collected via a validated self-administered questionnaire, ensuring that the sample cut across various administrative levels to capture a comprehensive view of digital maturity and its operational outcomes.

## Section two: The theoretical framework

## Literature review

### First: Digital maturity

**1.1 The Concept of Digital Maturity**

Profound changes across societies have been brought about by rapid developments in digital maturity. For instance, the integration of technology into business processes is believed to improve business efficiency, enhance organizational resilience, transform management practices, and improve value delivery to users. With the shift toward digitalization, the growth of available data has been staggering, enabling organizations to pinpoint strengths and weaknesses and act on corrections with minimal delay ( Ahmat et al., 2024: 3417). Gupta (2025: 5) notes that the provision of digital innovation and the advancement of modern technologies within institutionally beneficial yet consumer protective parameters are fundamental in advancing public and consumer interest policymaking. This is done through the creation of basic frameworks and general policies derived from societal value systems and orientations that help minimize the intangible and unintended adverse outcomes of rapid technological advancement. In addition, solving the issue of digital absence is not only about the digital divide, but also a matter of strategic importance in its own right, especially to enable small and medium enterprises (SMEs) in resource-dependent economies to function effectively in the transformations to come ( Sánchez-Ortiz et al., 2025: 13). Thus, digital maturity is mostly understood as the result of a process that is unfolding rather than a static ( Nielsen et al., 2024: 196).

Several definitions of digital maturity have been presented in the literature. Table 1 outlines the selected definitions in chronological order.

**Table 1.** Selected definitions of digital maturity in the literature.

No.	Researcher(s) and Year	Definition
1	( Nielsen, et al., 2024: 196)	The extent to which organizations are systematically prepared to continuously adapt to ongoing digital change.
2	( VAVURA & MATEI, 2024: 1115)	Helping organizations define a full integration goal for embedding digital tools and processes into the business model, achieving an ideal state of digitization.
3	( Kadoic, et al., 2024: 3)	A set of structured levels that define practices, processes, and organizational behaviors that reliably and sustainably produce desired outcomes. It measures an organization's capability for continuous improvement across specific dimensions until it reaches the desired level of maturity.
4	( Mulyandi, et al., 2025: 260)	The extent to which the received service meets customer expectations, reflecting their satisfaction and loyalty.

Source: Prepared by the Researchers.

The two researchers defined digital maturity “It is a measure of the organization’s capability of effectively adopting and utilizing digital technologies to advance its operations, provide value-adding services, promote innovations, and adapt to revolutions in the digital world. Incorporates the cultural aspect of technology, digital tools, structural organization, and the visions and perceptions of the employees and the top management of the organization into biotechnology.”

**1.2 Importance of Digital Maturity to Organizational Development**

Establishing the level of digital transformation maturity is of primary importance to the institution because it allows situational analysis and gap identification, which in turn allows the identification of the best suited transformation approach. In this step, organizational agility is reinforced, and the institution is positioned for sustainable long-term growth, especially when all dimensions of maturity are considered in the evaluation ( Haryanti et al. 2023, 2). Determining the level of digital maturity makes it possible to establish the difference between the actual and desired set of capabilities, and allows the institution to define strategic objectives. The underlying sophistication model continues to prove meaningful to organizations because its elements can be tailored to a variety of functions, including the assessment of goals, discretion, performance, and model transparency ( Merdin et al., 2023: 263). They continue to validate frameworks that facilitate the transition of services to digital mode while simultaneously lowering operational costs, enhancing productivity, and improving data and information quality ( Gerald et al., 2017). More than just enabling organizations to strategize and make plans to achieve a higher level of information age sophistication, digital maturity models provide a yardstick against which organizations may gauge their performance and make comparisons internally and externally ( Ochoa-Urrego & Peña, 2020:2). They strengthen productivity and competitiveness and help the top echelons of management articulate strategic goals ( Heidenwolf et al., 2025: 5902).

Tonder et al. (2024:112) accentuates the benefits of digital maturity models:

1. Wrap the organization’s skills and capabilities appraisal.
2. Elucidate the target level of maturity and the pathway to attaining it.
3. Access to other organizations for internal and external benchmarking.

Digitally mature organizations substantially outperform other organizations in their industry, boasting, on average, 6-9% more revenue per employee and 9-26% greater profitability than their competitors ( Thordsen & Bick, 2023: 967). They simplify the understanding of the organization’s level of digitization and enhance organizational competence and performance improvement ( Sándor & Ákos, 2022: 59).

**1.3 Dimensions of Digital Maturity**

As elaborated by the two researchers with respect to their individual views and spheres of activity, Digital Maturity goes beyond simpl technology; it includes managerial facets that reflect modifications to services, processes, skills, cultural aspects, and overall capabilities. At its most fundamental stage, digital maturity is confined to a continuous cycle of organizational learning that enables the firm to respond to the requirements of the hypercompetitive digital ecosystem ( Brodny & Tutak, 2021: 1-38). By review ing the existing literature, the authors isolated four fundamental dimensions through which an organization’s degree of digital maturity can be assessed. What follows is an elaborate account of each dimension in terms of its focus and importance.

1. **Technological Culture**
  Technological culture is the level that allows members of an organization to engage and function constructively and optimally with digital surroundings. This includes digital literacy, willingness to operate technological devices, and supportive behaviors toward innovation and change. The established culture is based on trust and transparency and nurtures multi disciplinary cooperation, which helps the organization in agility during digital transformation. By promoting a positive digital culture, the organization cultivates readiness for change that accelerates digital transformation ( Haryanti, 2023: 15–16).
2. **Technological Tools**
  The electronic infrastructure – devices, applications, and smart platforms – that are employed to facilitate operational activities, improve productivity, and provide a degree of automation are referred to as technological tools. These, however, are not just technical tools; the embodiments of untapped organizational wisdom and creativity are synthesized into technical tools that enhance organizational performance and quality. There is sufficient proof that successful digital endeavors are anchored to the appropriate understanding of the technical aspects and requisite resource provision. Thus, this dimension serves as an integral lever for the enduring digital growth ( Gill & VanBoskirk, 2016: 1-15).
3. **Organizational Structures**
  How tasks and functions are organized and the association of processes and relationships, both internally and externally, are referred to as organizational structures. It also serves as a pillar for digital governance. The more fluid and flexible these structures, the higher the organization’s ability to respond to market opportunities and challenges. Elements such as change management, organizational learning, and the deployment of data and analytics in decision processes fall within this dimension. Improved inter-departmental collaboration coupled with a sound organizational structure contributes to an agile system that is innovation-ready and able to respond to emerging opportunities and challenges expeditiously ( Tonder et al., 2024 & Al-Sabaawe et al., 2020).
4. **Visions and Perceptions**
  The delineation and comprehension of an organization’s visions and goals, as well as its level of digital objectives, is what the digital maturity framework calls the visions and perceptions strategic aspect. This includes the formulation of digital strategies that are legally, financially, and regulatory compliant. This also includes efficient management and channeling of digital investments toward value-adding endeavors. Outline and describe the elements of strategic focus, goals, and expectations of digital investments. State the importance of focus and value in digital investment. Focus as a success factor. Focus alters an organization’s success factors by reallocating its resources toward impactful and transformative digitization approaches ( Al-Sayabiya, 2022: 22–23).

### Second: E-Service quality

**2.1 The Concept of E-Service Quality**

Quality in dictionary form is defined as the nature, condition, or standard of something. The more abstract form of this definition, however, suggests that it does not speak of the specific dimensions of space and time. Quality is an indefinite and relevant phenomenon. Contemporary settings demand more focus and attention for achievement ( Afshari & Shokrollahi, 2025: 3). In this case, public service is the government’s responsibility to citizens, where it centers on regulation, control, offering services, and facilities for the sake of the public ( Julaeha et al., 2023: 3231). According to Mothey et al. (2025: 90), e-service quality refers to the delivery of products and services through the internet, smart devices, and digital channels. The quality of these services is defined by their ability to meet customer expectations in terms of their efficiency, reliability, security, and ease of use. E-service quality is typically measured by comparing customers’ expectations with the services they receive. This reflects the service’s ability to meet its needs. E-services represent an evolution of traditional services, and their quality is assessed based on the effectiveness and efficiency with which they are delivered via online platforms ( Utama & Trisnawati, 2024: 177 & Husien et al., 2019: 23).

The researchers present a selection of definitions and concepts from scholars and authors that reflect various intellectual and philosophical perspectives on the topic, as shown in Table 2 below:

**Table 2.** Selected definitions of E-service quality in the literature.

No.	Researcher(s) and Year	Definition
1	( Lisna, et al., 2024: 210)	Improving service quality through applications and the Internet.
2	( Ekasari, et al., 2024: 86)	An electronic service that provides customers with high cost benefits and time efficiency while facilitating the purchasing process.
3	( Lumbantobing, et al., 2025: 1936)	E-services delivered widely via the Internet to support customer activities efficiently and effectively.
4	( Pattanasing & Jiraphanumes, 2025: 2814)	A comprehensive customer evaluation and decision regarding the distinction and quality of e-service delivery in the virtual marketplace.
5	( Rinaldi, et al., 2025: 9684)	The extent to which a digital platform or IT-based service can effectively, easily, and reliably meet customer needs and expectations.

Source: Prepared by the Researchers.

The two researchers characterized the quality of electronic services as “the measuring of the efficiency and effectiveness of the services rendered through digital channels, in fulfilling user expectations, and in relation to the ease of use, security, reliability, responsiveness, and confidentiality.”

**2.2 Importance of E-Service Quality**

E-service quality is very important, especiall in the banking industry, because customers are able to receive services in a timely manner and improve operational efficiency, thus leading to higher customer satisfaction ( Asawawibul et al., 2025: 353). This is an indicator of the responsiveness of the banking system to a changing environment. E-service quality is crucial to the competitiveness of a digital business, and includes the level of responsiveness and ease of use of the system, system security, and overall system clarity. Increasing or obtaining customer satisfaction virtually guarantees customer loyalty ( Asawawibul et al., 2025). E-service quality also increases value perception and lowers costs and benefits from the use of AI and tracking systems to increase visibility and efficiency ( Asawawibul et al., 2025). These benefits include reduction of the costs associated with the systems low AI effectiveness at low levels in the system ( Asawawibul et al., 2025). The use of e-systems has proven capable of fulfilling their promises in multiple situations ( Aulawi et al., 2025: 92). E-service quality is also associated with customer satisfaction because the technology and new systems adopted help improve the efficiency of service delivery ( Rahahleh et al., 2020: 2760). Having any transaction anytime or anywhere through remote e-banking services helps reduce expenses while simultaneously enhancing the banking relationship with the customer ( Subahudin & Shahrom, 2023: 35–36). Loyalty is a function of customer satisfaction, and its quality is a source of competitive advantage and business sustainability ( Mohamed et al., 2025: 1056). E-banking also helps empower bank employees to offer more effective and efficient services, and customers to transact remotely, privately, and with a high degree of banking security ( Afshari & Shokrollahi, 2025: 1).

**2.3 Dimensions of E-Service Quality**

E-service quality dimensions, as defined by the two scholars, owe to the different approaches each takes to the dimensions relative to their areas of study. The sub-components or standards e-service quality dimensions stem from the decomposed overall construct into definable and comparable units. Theprimary dimensions include responsiveness, ease of use, confidentiality, trust and security, and reliability. Each dimension is defined by a certain set of indices. Disagreements among scholars on the definition of dimensions arise from differences in the theoretical perspective upon which the discussion is anchored, the type of industry, and the socio-technical environment. Some scholars, for example, add the dimensions of system efficiency, visualization, or personalization, while others opt to collapse redundant ( Mothey et al., 2025: 90).

In this case, the authors mentioned five distinct and validated dimensions of e-service quality and constructed them from previous literature, which are summarized as follows:

1. **Reliability**
  Concerns the e-service bank’s capacity to fulfill the e-service promised, meet the specific deadlines, and do it with the same accuracy each time. Systems are functional with only intermittent mistakes or breakdowns, and operations (e.g., transfer, bill payment, balance inquiries, etc.) are done right and done right the first time. It also pertains to having backup and restoration arrangements of service to ensure service non-stop service availability and protect customer confidence during peak or crisis periods ( Pattanasing & Jiraphanumes, 2025: 2814).
2. **Responsiveness**
  The rapid manner in which the bank responds to customer inquiries, complaints, and needs using technology (e.g., chat, email, phone, social media) with respect to rapid response times, ticketing, tracking, and practical/systematic swift resolution. It is covered by automatic answer systems and smart chatbots that resolve all the basic issues and push more complex issues to more specialized personnel. This ensures that there are no delays in the time taken to address and respond to customer needs ( Utama & Trisnawati, 2024: 177).
3. **Trust and Security**
  The servants and their services are protected by information security tools (encryption, Multiprotocol Label Switching [MPLS], and multi-factor authentic (MFA) authorization) and remain free from risks and uncertainties. This protection is monitored by privacy tools (transparency in privacy and usage policy, privacy and confidentiality in healthcare information, or other legal proofs for the claim), compliances (custom protection vs. legal protection, protected health information moderation, and legal compliance), and standards (legal) compliance ( Afshari & Shokrollahi, 2025). These documents contain data that can educate clients and improve their interactions in sophisticated safety cover environments. This framework also proves that funds, real estate, and even data exchanged in transactions (e.g., cryptocurrencies) have authentic transfer proofs. Such data increases tools, training, and customer wisdom for “smart” protection access guarantees, building trust, and confidence ( Thanasi-Boçe & Kulakli, 2023: 241).
4. **Ease of Use**
  The help environment, documents, and materials associated with the transaction can be coated and termed relief, self-and user explanatory support, and action pattern or flow support as a matrix or system of actions constituting the process. These documents and templates or outlines ensure that even learners with low or low operational skills can process transactions efficiently and with a guided structure. Actions become effortless and assisted in a matter of minutes ( Joyami & Salmani, 2019: 106).
5. **Confidentiality**
  The policy of data access and sharing is legible with minimal access points and moving barriers referring to or characterized by the least access sharing and disclosure in conjunction with data protection frames, regular data audits, secured access control, and data sharing restriction with authorization or legal foundation. Without any legal imprint, the confidentiality of a customer is the ideal and primary mode of a customer-banking relationship. Plans and notifications of the gathered customer data and their control sets clearly dictate usage ( Rahahleh et al., 2020: 2760).

## Section Third: The practical side of the study

### First: The study population

The study population for this research comprised managers and staff of the Al Rafidain Bank – Fallujah Branch because of their active participation in employing digital processes and providing clients with e-services. A purposive sampling method of choosing 102 members from the bank’s management and employees at different administrative and operational levels was used. The sample was cut across a variety of demographic characteristics such as sex, age, educational attainment, duration of employment, and circle of position in such a way as to mirror the bank’s structure. This will enable a better assessment of the association between a bank’s level of digital maturity and the quality of the electronic services offered.

Table 3 shows the distribution of the study sample by gender, where males represented 81.4% of the total sample, dominating over females, representing 18.6%. This indicates that males constituted the majority of the study population.

**Table 3.** Distribution of study sample by gender.

Percentage (%)	Frequency		
81.4%	83	**Male**	**Gender**
18.6%	19	**Female**	
**100.0%**	**102**	**Total**	

Table 4 shows the age-based stratification of study participants. The age cohort of 41-50 years comes first with 41.2%, the highest proportion among the benchmark sample categories. This suggests that most of the participants had ample professional experience and job stability. The 31-40 years age group comes next with 31.4%, indicative of moderately experienced and high-performing professionals. The 50 years and older group makes up 26.5% and is typically regarded as someone with extensive professional experience and considerable administrative and professional competencies. The less than 30 years group was the least represented with 1.0%, signaling the dearth of active youth participation in the study population.

**Table 4.** Distribution of the study sample by age group.

Percentage (%)	Frequency		
1.0%	1	**30 years or less**	** Age Group**
31.4%	32	**31 to 40 years**	
41.2%	42	**41 to 50 years**	
26.5%	27	**Over 50 years**	
**100.0%**	**102**	**Total**	

Table 5 shows the distribution of respondents according to their level of education. Most respondents resume bachelor degrees at the highest value of 71.6%. This means that the majority of respondents had sufficient education to undertake the administrative and technical tasks at hand. The second category, which pertains to secondary school qualifications, constituted 14.7%. This suggests that there is a segment of the study population that comprises employees with basic qualifications. We have respondents with higher diplomas accounting for 6.9%, postgraduate masters at 5.9%, and with the lowest value, doctorate holders at 1.0%. This suggests a scant new presence of highly scholarly employees in the sample domain. This, in turn, indicates a higher concentration on the routine than on scholarly or highly academic work in the case organization that is being investigated.

**Table 5.** Distribution of the study sample by educational qualification.

Percentage (%)	Frequency		
14.7%	15	**Secondary School**	**Educational Qualification**
71.6%	73	**Bachelor’s Degree**	
6.9%	7	**Higher Diploma**	
5.9%	6	**Master’s Degree**	
1.0%	1	**Doctorate Degree**	
**100.0%**	**102**	**Total**	

As shown in Table 6, the composition of this sample correlates with job titles. The largest group in this segment is the “other” category, with a 56.9% value. This suggests that operational and/or technical positions, which are not classified as higher administrative positions, were held by the majority of respondents. This is in line with the hierarchical structure of the organization, which has much membership in operational roles. The second largest group in the organization is classified as unit supervisors at 24.5%, which suggests that these employees have minimal supervisory responsibilities. Division supervisors represent 12.7% of the sample, while department managers have a very low rank of 5.9%. This indicates that the sample is dominated by low and middle administrative level positions compared to the small availability of sample members in high level administrative leadership positions.

**Table 6.** Distribution of the study sample by job position.

Percentage (%)	Frequency		
5.9%	6	**Department Manager**	**Job Position**
12.7%	13	**Division Head**	
24.5%	25	**Unit Supervisor**	
56.9%	58	**Other**	
**100.0%**	**102**	**Total**	

Table 7 demonstrates the sample’s distribution based on the years of service of each respondent. The figures indicate that the highest percentage per respondent is 33.3% in the 11 to 15 years category. This implies that a considerable number of respondents possess a moderate to substantial level of experience that allows them to efficiently understand the work environment and work requirements. The second highest percentage is 25.5% of respondents who have served for 26 years and above and are classified as long serving employees who are professionally stable and possess a well-developed understanding of the institution. The five years or less group holds 18.6%, in which there are a significant number of employees or even new recruits within the organization. The 16 20, 6 10, and 21 25 year age groups obtained 11.8%, 6.9%, and 3.9%, respectively. This indicates that the 21 to 25 years group had the lowest representation. These results indicate how the study population is temporally characterized by its diversity in relation to professional experience as well as a clear focus in the medium and above service years, suggesting organizational maturity and a well-developed base of institutional knowledge.

**Table 7.** Distribution of study sample according to years of service.

Percentage (%)	Frequency		
18.6%	19	**5 years or less**	** Years of Service**
6.9%	7	**6 to 10 years**	
33.3%	34	**11 to 15 years**	
11.8%	12	**16 to 20 years**	
3.9%	4	**21 to 25 years**	
25.5%	26	**More than 26 years**	
**100.0%**	**102**	**Total**	

This table ( Table 8) includes the study terms, variables, and dimensions, specifying the number of questions allocated to measure each dimension and its corresponding code. This classification aims to provide a clear methodological framework for measuring the main variables in the research.

**Table 8.** Terminology of the study variables and dimensions.

Number of questions	Code	Terminology and dimensions
5	TC	1. Technological Culture
5	TT	2. Technological Tools
5	OS	3. Organizational Structures
5	VP	4. Visions and Perceptions
20	DM	Digital Maturity
5	REL	1. Reliability
5	RES	2. Responsiveness
5	TOS	3. Trust and Security
5	EOU	4. Ease of Use
5	C	5. Confidentiality
25	ESQ	E-Service Quality

### Second: Testing the normal distribution of the data

In choosing the most suitable statistical approach for data analysis, the author focused on the normality of the distribution using Skewness and Kurtosis coefficients. The use of skewness and kurtosis as benchmarks is one of the first steps in statistical analysis to ascertain the degree of normality of a distribution. The distribution of values in skewness and kurtosis that range from (±1.96) depicts a lack of normality divergence more than normality at a significancelevel of 0.05. This enhances the sustenance of parametric methodologies, which are based on the normality of data and have higher power in determining relationships or differences among study variables. However, the more skewed or kurtotic the values are, the more they are not normal, and thus, the use of non-parametric methods is more appropriate. The statistical analysis results in Table 9 show that every value is contained in the result range. (±1.96) indicates a normal distribution and non-weakness in the degree of repetition of the data. This is a strong indication of the episode of unsound parametric statistical methods, which were not compiled in later analytical phases of the study. As a result, the data of the processes ensure that the primary assumption of the parametric methods used in these phases is fulfilled.

**Table 9.** Shows the results of the normality test for the research variables and dimensions.

Kurtosis	Skewness	Missing values	Sample size	Variables and dimensions
-0.952	-0.399	0	102	1. Technological Culture
-0.891	-0.061	0	102	2. Technological Tools
-0.709	-0.068	0	102	3. Organizational Structures
-0.152	-0.576	0	102	4. Visions and Perceptions
-0.697	-0.386	0	102	Digital Maturity
-0.893	-0.758	0	102	1. Reliability
-0.300	-0.677	0	102	2. Responsiveness
1.121	-0.690	0	102	3. Trust and Security
-0.493	-0.498	0	102	4. Ease of Use
-0.739	0.058	0	102	5. Confidentiality
-0.522	-0.488	0	102	E-Service Quality

The results of the statistical analysis presented in Table 9 indicate that all values fall within the acceptable range (±1.96), suggesting that the data are normally distributed and exhibit a high degree of consistency. This is a strong indication of the soundness of the data distribution and supports the use of parametric statistical methods in subsequent analytical stages, as one of their fundamental assumptions is fulfilled.

### Third: Reliability using split-half method

To measure the reliability of the instrument, the researcher used the Split-Half Reliability method, as shown in Table 10. The Spearman-Brown coefficient and the Guttman Split-Half coefficient both yielded a value of 0.839, indicating a high level of internal consistency. These results demonstrate that the instrument is reliable and suitable for use in the statistical analyses conducted in this study.

**Table 10.** Split-half reliability test.

Cronbach’s Alpha	Part 1	Value	.920
		N of Items	23
	Part 2	Value	.943
		N of Items	22
	Total N of Items		45
Correlation Between Forms			0.723
Spearman-Brown Coefficient	Equal Length		.839
	Unequal Length		0.839
Guttman Split-Half Coefficient			0.839

**Source**: SPSS V.28.

### Fourth: Sample adequacy

Analyzing the sample is the first step in factor analysis and can be performed using the KMO technique. If the value was greater than 0.50, the sample was considered appropriate for analysis. Moreover, correlations can be tested using Bartlett’s Test of Sphericity. If the P-value is less than 0.05, it means that the variables have significant relationships, thus confirming that the data are appropriate for factor analysis. These indicators are illustrated in more detail in the Results section, and the results are shown in Table 11.

**Table 11.** KMO and Bartlett’s test for the study scales.

Significance decision	Sig	Bartlett	Criterion	KMO	Dimensions
Significant	0.000	1663.370	Greater than 0.50	0.788	**Digital Maturity**
Significant	0.000	2691.022		0.842	**E-Service Quality**

The results from the analysis in the context of Table 11 show that the KMO values with respect to the variables (Digital Maturity and Quality of Electronic Services) were (0.788 and 0.842) respectively, well above the minimum 0.50 required in Kaiser’s classification. This suggests that the sample size was adequate and suitable for the analysis. In addition, Bartlett’s test results for the variables in the study were (1663.370 and 2691.022) respectively, and the level of significance was 0.000, which is much lower than the required 0.05. This means that the correlation matrix is not an identity matrix, and that the variables are interrelated to a considerable degree. Therefore, the sample size can be considered adequate, which is a good indicator for further statistical analysis.

### Fifth: Measurement quality assessment

**Research Model**

**Figure 2.**
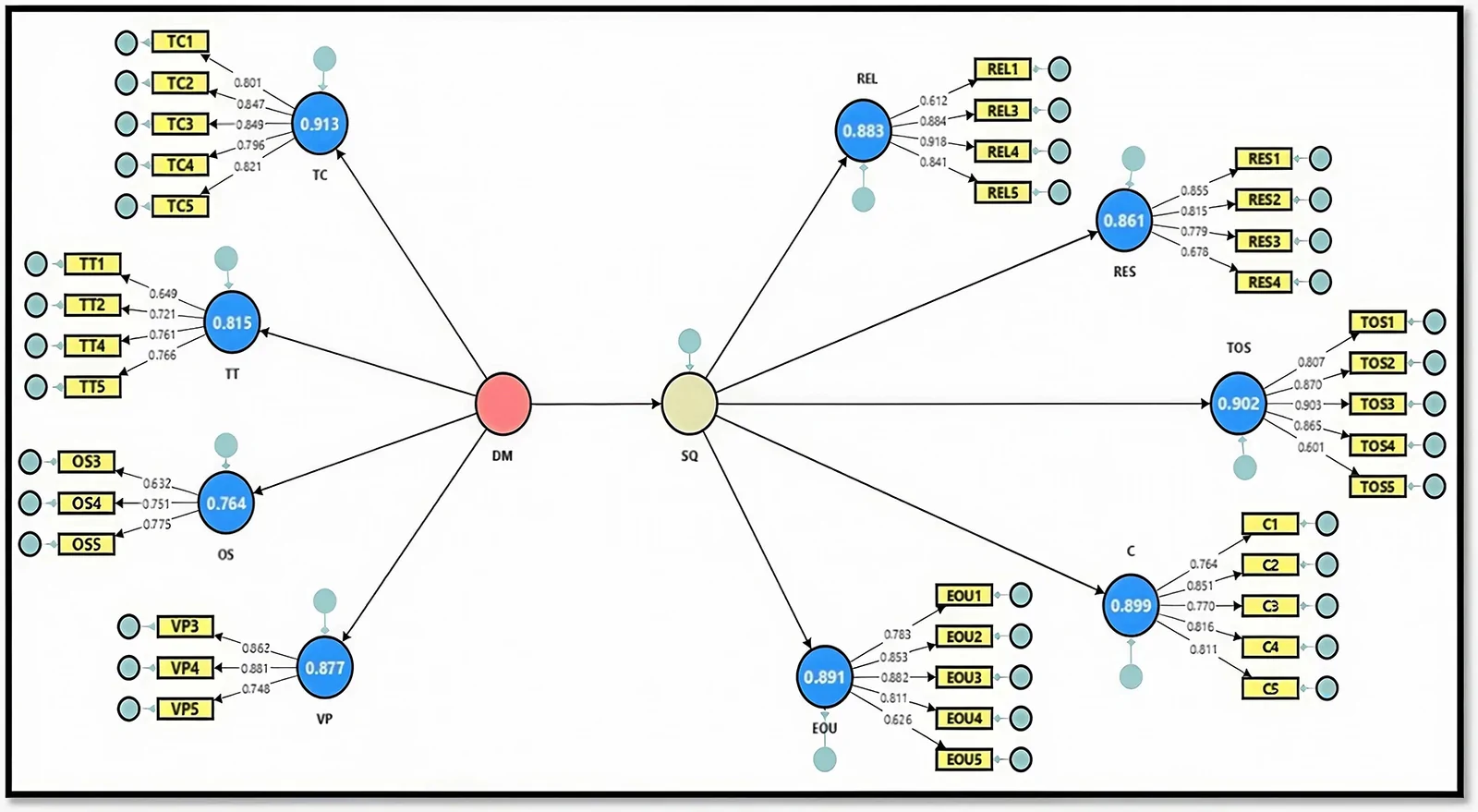
The complete research model. Source: Output from SmartPLS v.4 software. Note: The numbers within the circles represent Cronbach’s Alpha coefficients.

The previous chapter has shown us the results concerning testing both the validity and reliability of the dimensions concerning our research model. From Figure 2. The independent variable, Digital Maturity, has four major dimensions: TC, which represents Technological Culture, Technological Techniques (TT), Organizational Structures (OS), and vision and perceptions (VP) of a company. On the other hand, the dependent variable, Quality of Electronic Services, has five main dimensions: Reliability (REL), Responsiveness (RES), Trust and outspoken Security (TOS), Ease of Use (EOU), and Confidentiality (C). All the results presented in the table demonstrate satisfactory outcomes concerning Composite Reliability (CR), with a minimum threshold of 0.70. The ranges of 0.755 and 0.913 demonstrate an above-average internal consistency, along with the reliability of the measuring instruments. Most metrics suggest that uniform and non-uniform standardized alpha metrics suggest that the value of 0.70, which serves as a lower limit for standard metrics, was exceeded with single dimensions ranging from 0.757 to 0.913, proving the self-consistency responses of the dimensions, along with their outcome stability. The AVE results are also commendable. The set parameter in the AVE was 0.50, and the results of the metrics set were between 0.521 and 0.693, indicating satisfactory convergent validity. This suggests that the dimensions comprising each construct account for sufficient variances of their respective constructs; hence, one can confidently say that all reliability and validity measures remained within reasonable bounds, indicating that the quality of the scales and instruments used in the research were sufficient and that the research was trustworthy. Furthermore, the sub-dimensions of each variable sufficiently accounted for the total variance of the primary variables, which increased the validity of the analytical model in assessing the relationship between Digital Maturity and Quality of Electronic Services.

As illustrated in Table 12, the item factor loadings for the items were between (0.601), the lower limit, and (0.918), the upper limit, and all were above the accepted minimum cut off value of (0.40). This indicates that the items accurately represented the theoretical dimensions pertaining to them. Additionally, the (t) values ranged between (5.713) and (11.128) at a (P = 0.000) significance level, which is evidence that all relationships among items and dimensions are present and are sufficiently strong. These findings indicate a high level of construct and internal consistency, demonstrating the strong representation of the dimensions of digital maturity and e-service quality, and the high efficiency of the analytical model used in the research.

**Table 12.** Model fit indices.

Average variance extracted (AVE)	Composite reliability (rho_c)	Cronbach’s alpha (unstandardized)	Cronbach’s alpha (standardized)	Variables and dimensions
0.645	0.901	0.898	0.899	C
0.634	0.900	0.891	0.891	EOU
0.521	0.755	0.757	0.764	OS
0.676	0.900	0.884	0.883	REL
0.615	0.866	0.862	0.861	RES
0.678	0.913	0.913	0.913	TC
0.667	0.898	0.894	0.902	TOS
0.526	0.816	0.812	0.815	TT
0.693	0.868	0.877	0.877	VP
≥0.50	≥0.70	≥0.70	≥0.70	Criterion

### Sixth: Research variable for analysis is

1. **Digital Maturity**
Table 13 below shows the results of the descriptive analysis of digital maturity dimensions for the Rafidain Bank–Fallujah Branch. The overall mean of the results was (4.033) with a standard deviation of (0.585) and a coefficient of variation of (14.51%). This means that the branch was highly mature digitally, and the respondents had a good and fundamental understanding of the results. This demonstrates the management’s deep concern about the use of digital transformation technologies to improve banking operations and positively influence service quality. In the analysis of sub-dimensions, the tools and technologies dimension came first, and the mean was (4.061) with a coefficient of variation of (16.12%). This shows that the branch is equally devoted to the installation of modern technological systems and advanced digital tools to ease and improve banking and operational activities. The second is the organizational structure dimension with a mean of (3.856), which shows a fair level of team and unit administrative and organizational primary and secondary alignment to aid digital transformation processes. The third is the technological culture dimension with a mean of (4.104), which demonstrates that employees had a high level of digital awareness and skills, and that the practically executable skills were very low. ‘Vision and outlook’ data ranked 4th and has a mean of (4.111). This shows ambiguity in ‘digital transformation’ positively. Nevertheless, more detailed and realistic implementation plans are required. Overall, these results indicate that the Rafidain Bank – Fallujah Branch has a strong digital infrastructure that satisfies the prerequisites for digital transformation in’s aq the banking industry.
2. **E-Service Quality Variable**
  The descriptive level results for ‘Rafidain Bank – Fallujah Branch’ are shown in Table 13. The overall mean was (4.131) with a standard deviation of (0.543) and a coefficient of variation of 13.13%. This signifies a high level of e-service quality and respondents’ relative agreement. This variable ranked first among the principal variables of this investigation and suggests that the institution has a robust e-service infrastructure for user satisfaction. This promotes banking effectiveness. The increments where proactive was as follows, with a mean of (4.180) were noted as dominant through low variation (15.06). This suggests that digital interfaces and platforms in the bank are easily accessible to users. The results for the dimension of ‘confidentiality’ ranked second and has a more positive perception with a mean of (3.998) and a cv of (15.32%) on the scale of users’ trust in the bank’s capacity to safeguard personal and financial information. The dimension of ‘responsiveness’ ranked third with a more positive perception that has a mean of (4.154) and a cv of (15.36%) while claiming that the bank’s management and staff assist users in a satisfactory and timely manner via the bank’s digital channels. Next was the dimension of reliability, whose performance was perceived more positively and had a mean of (4.382) and a CV of (15.91%), which reflects the bank’s commitment to uninterrupted and accurate services.

Trust and assurance ranked fifth and had a mean of (3.939) and a CV of (17.60%), which suggests that employees are more relaxed when using the bank’s e-services. However, there is still a need to enhance measures to safeguard sensitive information and transactions from potential cyber threats.

**Table 13.** Descriptive statistics of the research variables and dimensions.

Ranking of dimensions and variables	Coefficient of variation	Std. deviation	Mean	Research variable dimensions
3	17.87	0.733	4.104	Technological Culture
1	16.12	0.655	4.061	Technological Tools
2	16.21	0.625	3.856	Organizational Structures
4	17.98	0.739	4.111	Visions and Outlook
Second	14.51	0.585	4.033	Digital Maturity
4	15.91	0.697	4.382	Reliability
3	15.36	0.638	4.154	Responsiveness
5	17.60	0.693	3.939	Trust and Security
1	15.06	0.630	4.180	Ease of Use
2	15.32	0.612	3.998	Confidentiality
First	13.13	0.543	4.131	E-Service Quality

**Source:** *Smart PLS 4 Software.*

### Seventh: Hypothesis testing

**1. Main Hypothesis One**

There is a statistically significant correlation between digital maturity and e-service quality.

**Figure 3.**
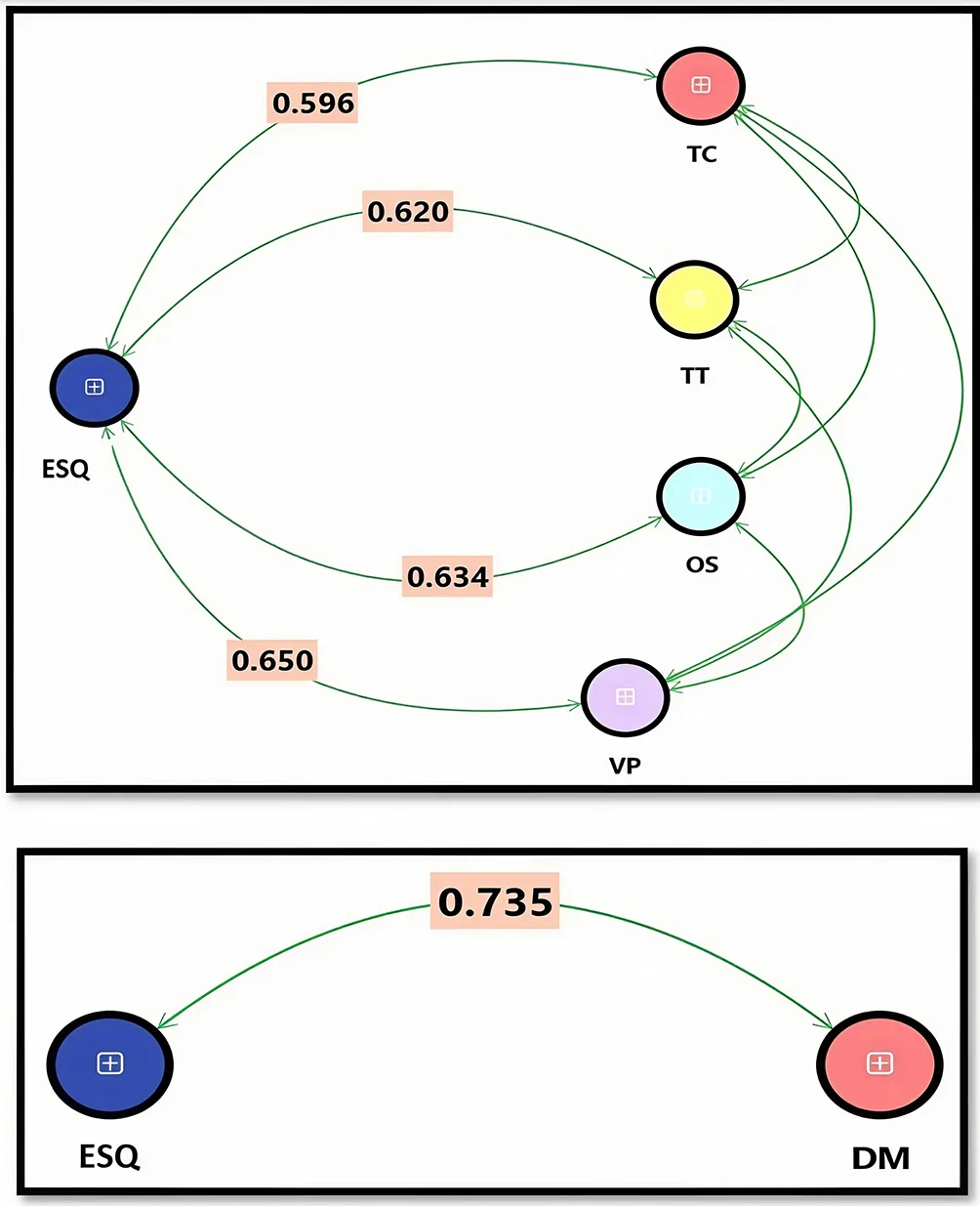
Correlation values between digital maturity dimensions and E-service quality.

In Table 14 and Figure 3, the outcomes of the correlation analysis between the variables Digital Maturity and E-Service Quality are presented. The results showed that the correlation coefficient was (0.735), indicating a strong positive relationship between the variables. Furthermore, the calculated Z-value was (9.348), which was above the threshold of (1.96) at a significance level of (P = 0.000). Therefore, the alternative hypothesis is retained: Digital Maturity and e-service quality are correlated and the correlation is significant.

**Table 14.** Correlation coefficients between digital maturity dimensions and E-Service quality.

Dependent variable	Sig	Z	(R)	Digital maturity dimensions
**E-Service Quality**	**0.000**	6.835	0.596	Technological Culture
	**0.000**	7.214	0.620	Technological Tools
	**0.000**	7.443	0.634	Organizational Structures
	**0.000**	7.714	0.650	Visions and Outlook
	**0.000**	9.348	0.735	Digital Maturity

**Source:** *Smart PLS 4 Software*.

The analysis clarifies that there is a positive correlation between Digital Maturity and E-Service Quality. This means that the greater the level of digital maturity in an organization, the greater the quality of the e-services offered. This suggests that an organization can achieve higher levels of digital culture, strong technological infrastructure, flexible organizational structures, and clear organizational visions to achieve an integrated balance between the technical and administrative levels. This would result in a higher operational efficiency, faster response, and better electronic service execution. Hence, digital maturity is an important factor in improving e-services by providing users with modern digital devices, advancing technological innovations, and enhancing user satisfaction with the digital services offered by the organization.

**a. Sub-Hypothesis One**

There is a statistically significant correlation between the technological culture dimension and e-service quality.

Tables 3 and 14 present the correlation analysis results between the “technological culture” dimension and the “quality of electronic services.” The correlation coefficient 0.596, indicating a strong positive relationship between the two variables. The calculated Z-value (6.835) exceeds the critical value (1.96) at a significance level of 0.000, which leads to acceptance of the alternative hypothesis.

This finding demonstrates that technological culture has a statistically significant correlation with quality of electronic services. This suggests that enhancing employees’ digital awareness and competencies directly contributes to improving the efficiency and quality of e-services and increasing beneficiary satisfaction.

**b. Sub-Hypothesis Two**

There is a statistically significant correlation between the technological tools dimension and e-service quality.

According to the data shown in Table 14 and Figure 3, there is a strong correlation between the “technological tools” dimension and the “quality of electronic services.” The correlation coefficient is 0.620, indicating a highly positive relationship. The calculated Z-value (7.214) was much greater than the critical value (1.96) at a significance level of 0.000, leading to the acceptance of the alternative hypothesis.

These results confirm the significant relationship between technological tools and e-service quality, emphasizing that the continuous development of digital systems and infrastructure plays a crucial role in enhancing service performance, efficiency, and user satisfaction.

**c. Sub-Hypothesis Three**

There is a statistically significant correlation between the organizational structures dimension and e-service quality.

Table 14 and Figure 3 display the correlation results between the “organizational structures” dimension and “quality of electronic services.” The correlation coefficient was 0.634, indicating a strong positive association. The calculated Z-value (7.443) surpassed the critical threshold (1.96) at the significance level of 0.000, confirming the acceptance of the alternative hypothesis.

These results reveal a statistically significant relationship between organizational structure and e-service quality. They indicated that flexible, well-structured, and digitally supportive administrative frameworks enhance institutional adaptability and contribute to higher service efficiency and quality.

**d. Sub-Hypothesis Four**

There is a statistically significant correlation between the *Visions and Outlook* dimension and e-service quality.

Table 14 and Figure 3 display the outcomes of the correlation assessment of Visions and Outlook, and E-Service Quality. The correlation coefficient of the two variables was 0.650, which was considered strong. The Z-value of 7.714 was greater than the critical value of 1.96, with a level of significance of P = 0.000. Therefore, the second hypothesis is supported. The correlation between the vision and outlook dimensions and e-service quality is statistically significant.

This means being able to visualize a service in digital form, and proactively looking into the construction of digital services also improves the effectiveness and quality of electronic services.

**2. Main Hypothesis Two**

There is a statistically significant effect of digital maturity on e-service quality.

**Figure 4.**
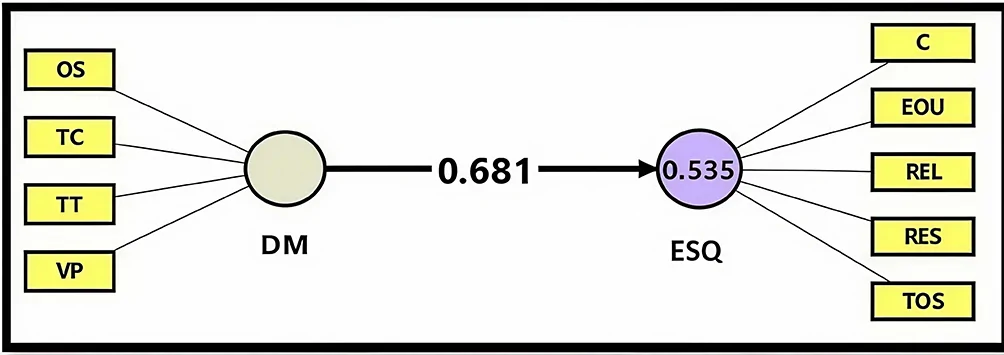
Testing the sub-hypotheses of digital maturity dimensions on E-service quality.

The calculated F-value associated with the influence of digital maturity on e-service quality, as presented in Table 15 and depicted in Figure 4, is (117.213). This figure is greater than the relevant critical F-value of (3.94) at a 0.05 significance level, thus strongly advocating the acceptance of the alternative hypothesis, which posits that digital maturity has a statistically significant effect on e-service quality. These results suggest that digital maturity can explain 53% of the variance in e-service quality. This suggests that institutions with greater digital maturity are more adept at integrating the technical and organizational infrastructure components necessary for the provision of high-quality services. In addition, the computed t-value for the digital maturity variable is (10.826), which is higher than the t-value of (1.984) at the significance level of 0.05, thus supporting the hypothesis that β is statistically different from zero. The regression coefficient (β = 0.681) suggests that an increase in digital maturity by one unit is associated with a 68% increase in the level of electronic services provided.

**Table 15.** Impact analysis of digital maturity dimensions on E-Service quality.

Dependent variable	Sig	(F)	(R ^2^) Adj	(R ^2^)	(R)	(t)	Digital maturity dimensions		
**E-Service Quality**	**0.000**	**55.138**	**0.349**	**0.355**	**0.596**	**9.372**	**2.321**	**(α)**	**Technological Culture**
						**7.426**	**0.441**	**(β)**	
	**0.000**	**62.447**	**0.378**	**0.384**	**0.620**	**7.647**	**2.045**	**(α)**	**Technological Tools**
						**7.902**	**0.514**	**(β)**	
	**0.000**	**67.247**	**0.396**	**0.402**	**0.634**	**7.660**	**2.008**	**(α)**	**Organizational Structures**
						**8.200**	**0.550**	**(β)**	
	**0.000**	**73.169**	**0.417**	**0.423**	**0.650**	**9.315**	**2.170**	**(α)**	**Visions and Perceptions**
						**8.554**	**0.477**	**(β)**	
	**0.000**	**117.213**	**0.535**	**0.540**	**0.735**	**5.404**	**1.385**	**(α)**	**Digital Maturity**
						**10.826**	**0.681**	**(β)**	

Chapter Four: Conclusions and Recommendations.

**a. Sub-Hypothesis One**

There is a statistically significant effect of the *Technological Culture* dimension on e-service quality.

**Figure 5.**
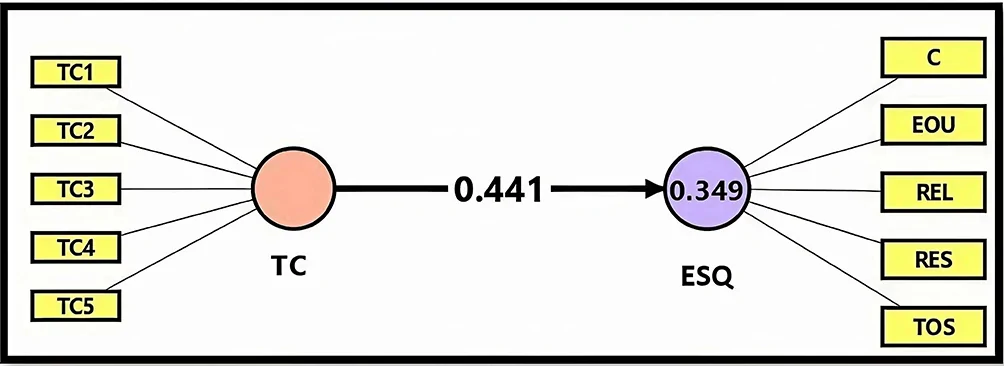
Effect analysis of the technological culture dimension on E-service quality.

These results further reinforce the notion that digital maturity acts as a primary element underlying an institution’s ability to improve its performance and undergo effective digital transformation. The value of the effect of technological culture on per-e-service value quality ( Table 15) and its quantification on the graph ( Figure 5) depict the obtained F-value, which stood at 55.138. This value exceeds the critical F-value (3.94 at the 0.05 significant level), which in turn provides sufficient support in favor of the acceptance of the alternative hypothesis, which states that there is an effect of the technological culture dimension on e-service quality and the effect is statistically significant. The findings show that technological culture accounts for 34 percent of the variance in e-service qualit, which indicates its importance in improving the efficiency of performance and advancement of digital services on offer. The extracted t-value for the technological culture variable was 7.426, which is much greater than the critical value of 1.984 at 0.05. This confirms the significance of β, which had a regression coefficient of 0.441. This finding implies that technological culture increases the level of e-service quality by 44 percent for every unit increase. Overall, these results prove that improving digital culture among employees enhances their e-performance efficiency, which also improves users’ overall experience.

**Figure 6.**
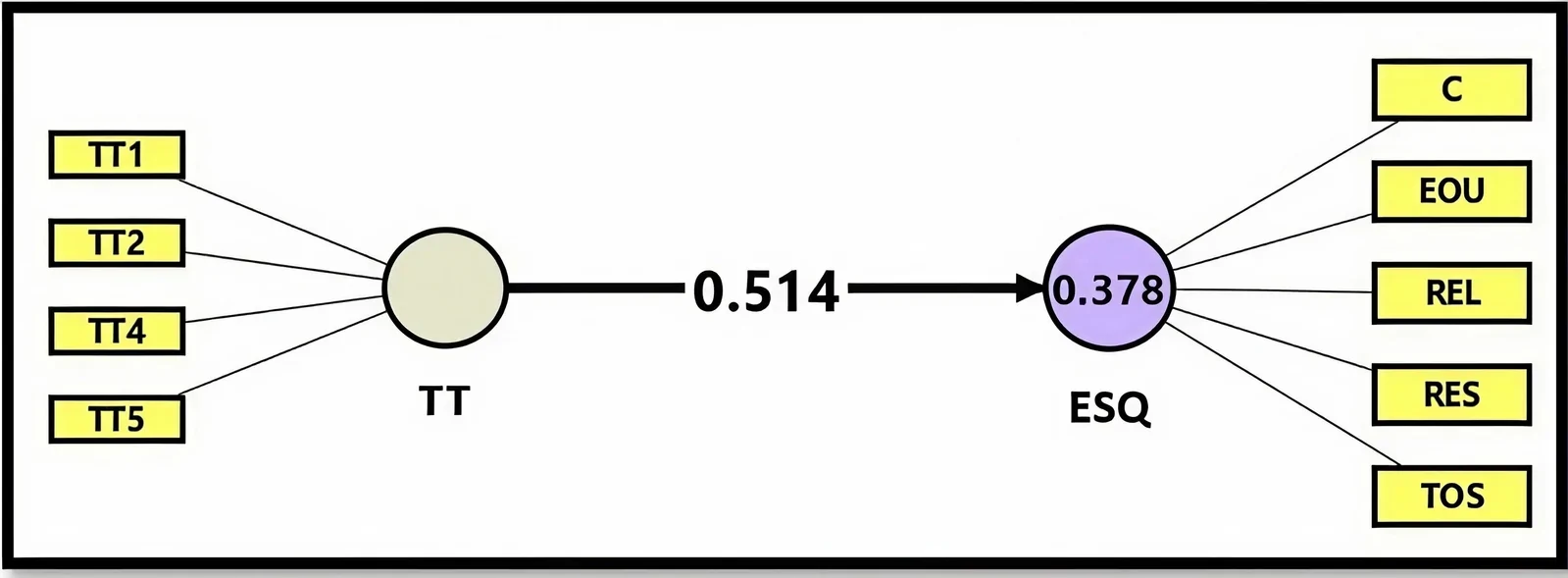
Effect analysis of the technological tools dimension on E-service quality.

As demonstrated in Table 15 and illustrated in Figure 6, the extracted F-value with respect to the impact of technological tools on e-service quality was (62.447). This is higher than the F critical value of (3.94) at the 0.05 significance level, thus providing adequate justification for the acceptance of the alternative hypothesis. This hypothesis suggests that the dimensions of technological tools have some impact on e-service quality.

**b. Sub-Hypothesis Two**

There is a statistically significant effect of the *Technological Tools* dimension on e-service quality.

The findings suggest that technological tools account for 37% of the variance in e-service quality, meaning that these tools are quite powerful in the context of system development and the improvement of digital infrastructure.

The extracted t-value corresponding to the technological tools was (7.902) and was also t-critical (1.984) at 0.05, thus confirming the significance of β. The regression coefficient (β = 0.514) suggests that the quality of e-services improved by 51% for every unit increase in the application of technological tools. This emphasizes the importance of digital tools in enhancing the operational efficiency and overall performance of the institution.

**c. Sub-Hypothesis Three**

There is a statistically significant effect of the *Organizational Structures* dimension on e-service quality.

**Figure 7.**
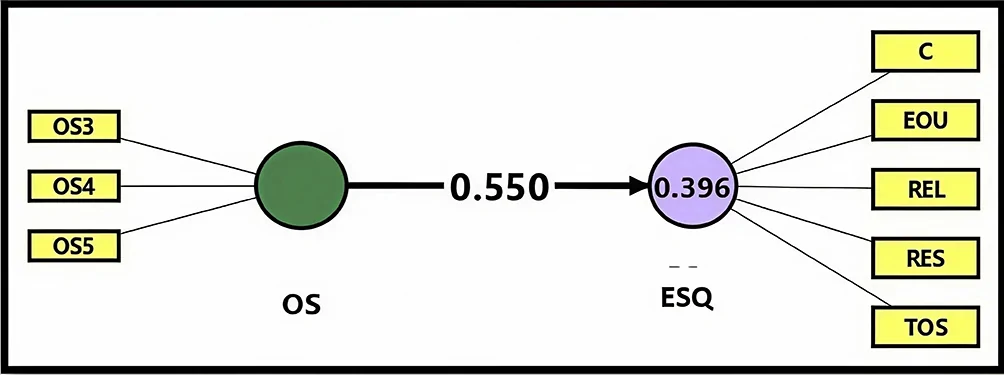
Effect analysis of the organizational structures dimension on E-service quality.

Table 15 and Figure 7 show the extracted F-value for the effect of *organizational structures* on *e-service quality*, which was (67.247). This value is greater than the critical F-value of (3.94) at the significance level of 0.05, providing sufficient support for accepting the alternative hypothesis that states that *There is a statistically significant effect of the organizational structures dimension on e-service quality.* The results indicate that *organizational structures* explain 39% of the variance in e-service quality, reflecting their effectiveness in organizing the roles and responsibilities that support digital transformation. Moreover, the extracted t-value for the organizational structures variable was (8.200), which exceeded the critical value of (1.984) at a level of 0.05, confirming the significance of β. The regression coefficient was (β = 0.550), indicating that a one-unit increase in organizational structure leads to a 55% improvement in the quality of e-services.

This underscores the enhancement of organizational effort integration in supporting digital transformation and improving service efficiency.

**d. Sub-Hypothesis Four**

There is a statistically significant effect of the *Visions and Perceptions* dimension on e-service quality.

**Figure 8.**
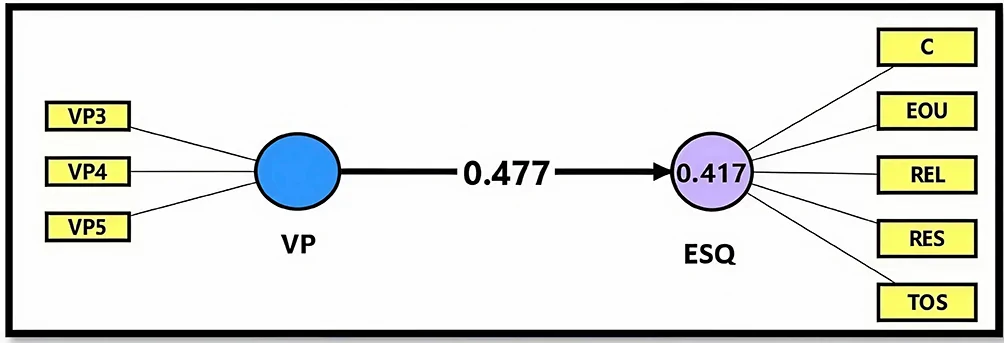
Effect analysis of the visions and perceptions dimension on E-service quality.

Based on the findings, technological tools are responsible for 37% of the variance in e-service quality, indicating that they are very effective in the context of system development and the enhancement of digital infrastructure. For the technological tools, the estimated t-value was (7.902) and t-critical was (1.984) at 0.05, which confirms the significance of β. The regression coefficient (β = 0.514) indicates that e-service quality improves with an increase in technological tool application by 51% for every unit increase. This further reinforces the effectiveness of technological tools in operationalizing institutions and improving productivity. The calculated F-value of the influence of visions and perceptions on e-service quality is (73.169), as presented in Table 15 and illustrated in Figure 8. This value exceeds the critical F-value of (3.94) at a significance level of 0.05. Thus, there is ample evidence to support the alternate hypothesis that claims there is an influence and that the visions and perceptions dimension has an influence on e-service quality. The data show that visions and perceptions account for 41% of the change in e-service quality, which demonstrates how much they cansteer digital change and foster more anticipative thinking about technology use. The t-value thatpertains to visions and perceptions is (8.554). Similar to the previous t-value discussed, this value is more than the 0.05 critical value of (1.984), which further lets us conclude that the hypothesis that β = 0 may be rejected. The value of the regression coefficient is (β = 0.477). This implies that an increase in vision and perception leads to an increase in e-service quality, and in this case, 47%. This result illustrates that a well-defined digital vision will manifest in enhanced organizational performance and service quality.

## First: Results

1. Results: The outcomes of the hypotheses are summarized as follows: 1. The results showed a strong and statistically significant correlation between digital maturity and the quality of electronic services (correlation coefficient = 0.735, Z = 9.348), in dicating that advanced levels of digital maturity improve the quality of electronic services in terms of better technical and administrative integration, personnel empowerment, enhanced performance efficiency, and beneficiary satisfaction.
2. The results revealed that digital maturity is a strong predictor of the quality of electronic services, accounting for 53% of the variance. The results (F = 117.213) and (β = 0.681) indicate that a unit increase in digital maturity brings about a 68% increase in service quality, thereby reinforcing the notion that digital maturity is a vital component in attaining high levels of digital performance and the transformation of institutions.
3. The path analysis results provide evidence that the quality of electronic services at the Rafidain Bank – Fallujah Branch is influenced by three dimensions of digital maturity: technological culture, organizational structures, and visions and perceptions. In contrast, technological tools had no meaningful impact, reinforcing the idea that digital maturity is more than the presence of technology; it integrates into an adaptable organizational structure with a well-defined vision and nurturing digital culture. Collectively these dimensions accounted for nearly 56% of the variability in the quality of the electronic services.

## Second: Conclusions

1. From the analysis, the level of digital maturity at the Rafidain Bank – Fallujah Branch was considered to be high, which means the bank is well on its way to digital transformation and effectively utilizes technical systems and digital infrastructure to automate its daily banking processes.
2. The data indicate that the dimension of technological tools was the most advanced in the scale of digital maturity, which is indicative of the bank's investment in the development of technical systems and the integration of sophisticated digital instruments aimed at enhancing operational efficiency and service delivery to customers.
3. This dimension of culture was quite favorable and signified growing levels of digital awareness, although there was a gap in the ability to establish the transformative culture within the workplace, which needed specialized training for the adoption of Innovation and Digital skills.
4. The good level of the organizational structure dimension shows that banks administrative settings are suited for the application of digital transformation. However, more flexibility in the allocation of tasks and authorities must be adopted for rapid decision making pertaining to digital issues.
5. The positive level of the vision and perception dimension suggests that digital service enhancement activities are approached with a vision. However, sensible complete integration poses a challenge in the absence of precise actionable strategies.
6. The results showed that the quality of e-services recorded the highest level among the study variables, confirming the bank’s success in delivering stable, user-friendly, and secure electronic services that enhance customer satisfaction and strengthen its position in the digital banking market.
7. The correlation analysis results demonstrated a strong and statistically significant relationship between digital maturity and the quality of e-services, indicating that the advancement of the bank’s digital infrastructure leads to improvements in the quality of electronic services provided to customers.
8. The findings confirmed that each dimension of digital maturity (culture, technology, organizational structure, and vision) has a statistically significant impact on the quality of e-services, emphasizing that digital maturity is an integrated system that cannot be achieved without the alignment of all its components.
9. The results indicated that digital maturity explains more than half of the variance in the quality of e-services, highlighting its strategic role in improving banking performance and enhancing the institution’s competitiveness in an increasingly digital banking environment.
10. The final results showed that the greatest impact on the quality of e-services stems from the dimensions of technological culture, organizational structure, and digital vision, whereas the impact of technological infrastructure alone is less significant when not supported by adequate managerial activation and training.

### Third: Recommendations

1. The bank’s management should persist in the support of projects on digital transformation and broaden them to all services and banking operations, deepening the digitalization of the inter-departmental integration of all bank units.
2. Foster technological culture by training and development to increase the capacity of employees to modern systems and encourage practitioners to professionally innovate and resolve technical issues.
3. Invest in continual modernization of technology, in compliance with international cyber protection of banking information, to facilitate customer confidence in banking systems technology.
4. Support the dissolution of traditional hierarchies and replace them with looser frameworks that promote collaborative workflows between in-person and remote work coupled with shifting responsibilities to meet the realities of emerging technologies.
5. Revise the bank’s vague goals and assign cross-functional execution and measurement to improve the operationalization and longevity of the objectives in the digital transformation plan.
6. Greater investment in cybersecurity is necessary, including the adoption of advanced protection systems against cyber threats and breaches, thereby reinforcing trust and security within the e-service environment.
7. Continuous customer engagement programs should be activated to better understand their digital needs and expectations, enabling the development of e-services that are more aligned with user preferences and improve the overall banking experience.
8. The bank should adopt incentive policies for employees working in digital domains to foster innovation and increase motivation toward adopting new technological solutions in daily operations.
9. The findings underscore the importance of collaboration between technical and administrative departments to unify efforts toward achieving full digital transformation and ensuring integration between technology and operational processes.
10. It is recommended that the bank focus on strengthening technological culture, enhancing organizational flexibility, and clarifying its digital vision, alongside providing effective training and managerial support, to ensure optimal utilization of technological infrastructure in improving e-service quality.

## Informed consent

Written informed consent was obtained from all individual participants prior to their participation in the study. All participants were informed about the purpose of the research, their right to withdraw at any time, and the confidentiality of their data.

## Ethical approval

This study received ethical approval from the Scientific Research Ethics Committee of the Scientific Affairs Unit at the University of Fallujah. All research procedures were conducted in accordance with the approved ethical standards, ensuring the protection of participants’ rights and the confidentiality of their information. The ethical approval was formally granted under the reference code **UOF.HUM.2025.001**.

## Data Availability

The data supporting the findings of this study are openly available in [Zenodo] at [ https://doi.org/10.5281/zenodo.17875057], under the Creative Commons Attribution 4.0 International (CC BY 4.0) license. ( Turki & Mahmood, 2025) Repository name: [The Role of Digital Maturity in Achieving the Quality of E-Services: An Analytical Study at Al-Rafidain Bank – Fallujah Branch]. [ https://doi.org/10.5281/zenodo.17875057]. ( Turki & Mahmood, 2025) The project contains the following underlying data: [Researcher Younes’s database.xlsx] (Raw numerical data used for the main statistical analysis). Repository name: [The Role of Digital Maturity in Achieving the Quality of E-Services: An Analytical Study at Al-Rafidain Bank – Fallujah Branch]. [ https://doi.org/10.5281/zenodo.17875057]. ( Turki & Mahmood, 2025) This project contains the following extended data: [Questionnaire Form.docx] (The questionnaire used for data collection). Data are available under the terms of the Creative Commons Attribution 4.0 International (CC BY 4.0). This study is an observational survey-based research and follows the STROBE reporting guidelines. No CONSORT or ARRIVE checklists are required as the study does not involve clinical trials or animal experiments.
