# Supplementary material for: The Role of Digital Maturity in Achieving the Quality of E-Services: An Analytical Study at Al-Rafidain Bank – Fallujah Branch
Source: F1000Res. 2026 Jul 27;15:157. Originally published 2026 Jan 31. [Version 2] doi: 10.12688/f1000research.174203.2 (PMC13559999; doi:10.12688/f1000research.174203.2)
Supplement: Peer review history

# Reviewer report: Reviewer response for version 2

**DOI:** 10.5256/f1000research.205779.r505397

**Published:** 2026-08-12

**Authors:** Ighomereho O. Salome

**Affiliations:** 1 Redeemer’s University, Osun State, Nigeria

l have checked my reservations vis-a-vis the corrected version of the article and l am satisfied with the amendments. 

 l therefore approve the article for indexing.

Is the work clearly and accurately presented and does it cite the current literature?

Yes

If applicable, is the statistical analysis and its interpretation appropriate?

Yes

Are all the source data underlying the results available to ensure full reproducibility?

Yes

Is the study design appropriate and is the work technically sound?

Partly

Are the conclusions drawn adequately supported by the results?

Partly

Are sufficient details of methods and analysis provided to allow replication by others?

Partly

Reviewer Expertise:

Service Quality

I confirm that I have read this submission and believe that I have an appropriate level of expertise to confirm that it is of an acceptable scientific standard.

# Reviewer report: Reviewer response for version 2

**DOI:** 10.5256/f1000research.205779.r505398

**Published:** 2026-07-28

**Authors:** Malek Al-Edenat

**Affiliations:** 1 Department of Business, College of Business, Tafila Technical University, Tafila, Jordan

I'm satisfied with the amendments made

Is the work clearly and accurately presented and does it cite the current literature?

Yes

If applicable, is the statistical analysis and its interpretation appropriate?

Yes

Are all the source data underlying the results available to ensure full reproducibility?

Yes

Is the study design appropriate and is the work technically sound?

Yes

Are the conclusions drawn adequately supported by the results?

Yes

Are sufficient details of methods and analysis provided to allow replication by others?

Yes

Reviewer Expertise:

Leadership, Transformation, Performance

I confirm that I have read this submission and believe that I have an appropriate level of expertise to confirm that it is of an acceptable scientific standard.

# Reviewer report: Reviewer response for version 1

**DOI:** 10.5256/f1000research.192085.r481732

**Published:** 2026-07-07

**Authors:** Malek Al-Edenat

**Affiliations:** 1 Department of Business, College of Business, Tafila Technical University, Tafila, Jordan

1. **Theoretical Framework – Missing Explicit Theory**

- The abstract mentions “Theoretical/Framework” but no specific theory is named or discussed.
- **Suggestion:** Explicitly state the underpinning theory (e.g., Technology Acceptance Model, TOE Framework, or Digital Maturity Models). Discuss it in the literature review, not just in the abstract.

2. **Problem Statement – Specificity Lacking**

- The research problem is described generally (“digital service gap in several Iraqi banks”).
- **Suggestion:** Add 2–3 concrete problems observed at *Al-Rafidain Bank – Fallujah Branch* (e.g., system downtime, low customer adoption, slow complaint resolution) to justify the case study.

3. **Sampling Method Mislabeling**

- Page 9 states “purposive sampling” but page 10–11 uses “stratified” language (e.g., “stratified across demographic characteristics”).
- **Suggestion:** Revise wording (as the peer reviewer noted) to “the sample cut across demographic characteristics” unless true stratification was used.

4. **Inconsistent Hypothesis Wording**

- Main Hypothesis One (H1) uses *correlation*, but Main Hypothesis Two (H2) uses *impact*. Later, “affect” appears interchangeably with “impact.”
- **Suggestion:** Stick to one term per hypothesis type. For regression/β, use “impact.” For Pearson/Z, use “correlation.”

5. **Contradiction in Technological Tools Findings**

- Abstract & page 20: “impact of technological tools was weak.”
- But Table 15 & page 19 show statistically significant impact (β = 0.514, t = 7.902, p < 0.001), explaining 37% of variance.
- **Suggestion:** Reconcile this contradiction. The text says “no meaningful impact” but the statistics indicate strong significance. Perhaps you mean “weaker relative to other dimensions”? Clarify.

6. **Minor Typographical & Formatting Issues**

- Page 3: “ease of invective” → should be “ease of use” (typo).
- Page 4, 7, 8: Inconsistent numbering of sections (e.g., “Section one” vs “1.”).
- Page 23–24: References include duplicate entries (Husien et al., 2019 appears twice).
- Table 4: “31 to 40 years” repeated in age group labels.

7. **Conclusions – Some Claims Not Directly Supported by Data**

- Page 20: “delay in administrative decision-making due to centralization” – this finding is plausible but not directly measured in the questionnaire. It appears to come from authors’ interpretation rather than direct items.
- **Suggestion:** Either add a questionnaire item or rephrase as “authors’ inference from qualitative insights.”

8. **Recommendations – Slightly Generic**

- Recommendations (e.g., “train employees,” “upgrade infrastructure”) are valid but common.
- **Suggestion:** Prioritize recommendations based on the dimensions that had strongest impact (culture, structures, vision). Make them more specific to Fallujah branch context.

Is the work clearly and accurately presented and does it cite the current literature?

Yes

If applicable, is the statistical analysis and its interpretation appropriate?

Yes

Are all the source data underlying the results available to ensure full reproducibility?

Yes

Is the study design appropriate and is the work technically sound?

Yes

Are the conclusions drawn adequately supported by the results?

Yes

Are sufficient details of methods and analysis provided to allow replication by others?

Yes

Reviewer Expertise:

Leadership, Transformation, Performance

I confirm that I have read this submission and believe that I have an appropriate level of expertise to confirm that it is of an acceptable scientific standard.

## References

[1] : Organizational competencies toward digital transformation at the events of disruptive changes: an operational process innovation perspective. *Competitiveness Review: An International Business Journal* .2023;33(4) : 10.1108/CR-05-2021-0081690-710 10.1108/CR-05-2021-0081

# Response

**Published:** 2026-07-07

**Authors:** Younis akram

**Affiliations:** General Administration, University of Fallujah, Al-Fallujah, Al Anbar Governorate, Iraq

​Dear Dr. Malek Al-Edenat, ​We would like to express our profound gratitude for your time, effort, and highly constructive evaluation of our manuscript. Your precise recommendations have been instrumental in elevating the academic rigor and structural clarity of our study. ​We have meticulously and comprehensively addressed all of your recommendations in the newly revised version submitted to the journal, as detailed below:

 Action Taken on Theoretical Framework: We have explicitly adopted the Unified Theory of Acceptance and Use of Technology (UTAUT2) as the underpinning theoretical framework for this study. This theory has been highlighted in the Abstract and fully discussed in a newly added, comprehensive subsection within the Literature Review (Section 2.1) to bridge the theoretical foundation with our study's variables. ​

 Action Taken on Problem Statement: The problem statement has been substantially sharpened and localized to provide a stronger justification for the case study. We have explicitly incorporated 3 concrete operational challenges observed at the Al-Rafidain Bank – Fallujah Branch: frequent core system downtime, low customer adaptation rates to mobile banking applications, and delayed response times in resolving online customer complaints. ​

 Action Taken on Sampling Method: Since true statistical stratification was not utilized, we have entirely removed the word "stratified" from pages 9–11 to avoid mislabeling. In strict accordance with your suggestion, the text has been revised to state: "the sample cut across demographic characteristics," accurately preserving the true purposive sampling design of the research.

 ​Action Taken on Hypothesis Wording: We have thoroughly reviewed and standardized the terminology throughout the entire manuscript. The term "correlation" is now strictly and exclusively used for Pearson/Z statistical tests, while the term "impact" is used for regression and \beta-coefficient analyses. All loose or interchangeable terms like "affect" have been eliminated for strict scientific consistency.

 ​Action Taken on Findings Contradiction: This textual oversight has been fully resolved to match our statistical reality. The discussion in the Abstract and on page 20 has been corrected to explicitly state that the "Technological Tools" dimension has a statistically significant and strong impact on e-service quality (\beta = 0.514, p < 0.001), but notes that it was relatively weaker when compared to other organizational dimensions (such as culture and vision) within the branch.

 Action Taken on Typographical & Formatting Issues: All formatting and editing issues have been resolved. The typographical error on Page 3 has been corrected to "ease of use." Section numbering has been completely standardized using uniform decimal notation (e.g., 1., 1.1, 1.2) across all chapters. The duplicate reference entry for Husien et al. (2019) has been removed, and the repetitive age group labels in Table 4 have been corrected.

 ​Action Taken on Conclusions: Since administrative centralization was not quantitatively measured via the questionnaire items, we have rephrased this conclusion exactly as suggested. It is now explicitly presented as the "authors' inference derived from qualitative insights and field observations" during the data collection process, rather than a direct quantitative finding. ​

 Action Taken on Recommendations: We have completely restructured the recommendations section, moving away from generic tips to focus directly on our highest-impact dimensions (Culture and Vision). The new recommendations are highly specific to the Al-Rafidain Bank – Fallujah Branch context, emphasizing localized digital cultural transformation, lowering resistance to fintech change, and empowering the branch with agile decision-making. ​We believe that these extensive updates have thoroughly fulfilled your recommendations and significantly bolstered the scholarly value of our paper. The updated manuscript has been submitted to the editorial platform for your final review. ​Thank you once again for your valuable guidance. ​Best regards, ​Younis Akram Turki Corresponding Author Fallujah, Iraq

# Reviewer report: Reviewer response for version 1

**DOI:** 10.5256/f1000research.192085.r463032

**Published:** 2026-03-25

**Authors:** Ighomereho O. Salome

**Affiliations:** 1 Redeemer’s University, Osun State, Nigeria

Sections Report are as follow:

 1. Under theoretical framework in abstract, indicate the theory underpinning the study. This should also be discussed in the literature review.

 2. The background is well presented with evidence of recent citations of 2025.

 3. Under problem statement, the authors should add some of the problems in Al-Rafidain Bank that captured their interest to study the bank.

 4. Use alphabetical numbering for research significance and research objectives for consistency with research hypotheses numbering. Again, the section heading is Arabic numbering.

 5. The literature review addressed the concepts in the study adequately but the theory underpinning the study was not discussed.

 6. The methodology is not detailed enough. Provide a full report of the methods that you used and the justification for your choice. 

 7. The sentence in line 4 under study population should be rephrased. The word stratified should be removed because there is no evidence of stratified sampling. I suggest the sentence should read " the sample cut across"

 8. For the explanation of Table 6, instead of the largest segment in this segment, it should read the largest group in this segment

 9. Under reliability, confirm if the value for Spearman-Brown coefficient is the same as Guttman Split-Half. The values are the same, but you constructed the sentence as if the values are different.

 10. In restating the hypotheses under hypotheses testing, state them as they were presented in the introductory section. You set out to test impact, but when you got to analysis, you changed to affect. I suggest you stick to impact.

 11. Revisit the conclusion and recommendations. Anyone outside the findings of the study should be deleted.

Is the work clearly and accurately presented and does it cite the current literature?

Yes

If applicable, is the statistical analysis and its interpretation appropriate?

Yes

Are all the source data underlying the results available to ensure full reproducibility?

Yes

Is the study design appropriate and is the work technically sound?

Partly

Are the conclusions drawn adequately supported by the results?

Partly

Are sufficient details of methods and analysis provided to allow replication by others?

Partly

Reviewer Expertise:

Service Quality

I confirm that I have read this submission and believe that I have an appropriate level of expertise to confirm that it is of an acceptable scientific standard, however I have significant reservations, as outlined above.

# Response

**Published:** 2026-04-03

**Authors:** Younis akram

**Affiliations:** General Administration, University of Fallujah, Al-Fallujah, Al Anbar Governorate, Iraq

​Dear Dr. Ighomereho O. Salome, ​I would like to express my sincere gratitude and appreciation for the time and effort you invested in reviewing my manuscript. Your insightful comments and constructive feedback have been invaluable in enhancing the academic quality and methodological rigor of this study.

   ​I am pleased to inform you that I have meticulously addressed all the points raised in your report as follows: ​Theoretical Framework: I have explicitly incorporated the Extended Unified Theory of Acceptance and Use of Technology (UTAUT2) within both the abstract and the literature review, clarifying its role as the theoretical underpinning of the study. ​Problem Statement: The section has been enriched with specific challenges faced by Al-Rafidain Bank to provide a more concrete practical context. ​Methodology & Sampling: I have expanded the methodology section with a detailed report and justification. Furthermore, I have revised the sampling description to "the sample cut across" various levels, as per your excellent suggestion. ​Consistency & Terminology: All numbering has been standardized to alphabetical format where requested, and the term "Impact" has been used consistently throughout the manuscript to align with the research hypotheses. ​Reliability & Tables: I have corrected the phrasing regarding the Spearman-Brown and Guttman Split-Half coefficients and updated the terminology in Table 6 to "largest group." ​Conclusion: The conclusions and recommendations have been strictly revisited to ensure they are directly supported by the study’s empirical findings. ​Thank you once again for your professional guidance, which has significantly strengthened this work. I look forward to your further feedback on this revised version.

   ​Kind regards, ​Younis Akram Turki University of Fallujah
